# A Gas Sensing Channel Composited with Pristine and Oxygen Plasma-Treated Graphene

**DOI:** 10.3390/s19030625

**Published:** 2019-02-01

**Authors:** Haiyang Wu, Xiangrui Bu, Minming Deng, Guangbing Chen, Guohe Zhang, Xin Li, Xiaoli Wang, Weihua Liu

**Affiliations:** 1 School of Microelectronics, School of Electronics and Information Engineering, Xi’an Jiaotong University, Xi’an 710049, China; wuhaiyang@stu.xjtu.edu.cn (H.W.); bxr1212@stu.xjtu.edu.cn (X.B.); zhangguohe@xjtu.edu.cn (G.Z.); lx@mail.xjtu.edu.cn (X.L.); xlwang@mail.xjtu.edu.cn (X.W.); 2 Science and Technology on Analog Integrated Circuit Laboratory, Chongqing 401332, China; yangjing@cetccq.com.cn (M.D.); cgb@sisc.com.cn (G.C.); 3 Guangdong Shunde Xi’an Jiaotong University Academy, NO.3 Deshengdong Road, Daliang, Shunde District, Foshan 528300, China; 4 School of Science, Xi’an Jiaotong University, Xi’an 710049, China; 5 Key Laboratory for Physical Electronics and Devices of the Ministry of Education, Department of Electronic Science and Technology, School of Electronic and Information Engineering, Xi’an Jiaotong University, 28 West Xianning Road, Xi’an 710049, China; 6 Research institute of Xi’an Jiaotong University (Zhejiang), Hangzhou, Zhejiang 311215, China

**Keywords:** graphene, oxygen plasma treatment, gas sensor, NH_3_

## Abstract

Oxygen plasma treatment has been reported as an effective way of improving the response of graphene gas sensors. In this work, a gas sensor based on a composite graphene channel with a layer of pristine graphene (G) at the bottom and an oxygen plasma-treated graphene (OP-G) as a covering layer was reported. The OP-G on top provided oxygen functional groups and serves as the gas molecule grippers, while the as-grown graphene beneath serves as a fast carrier transport path. Thus, the composite channel (OP-G/G) demonstrated significantly improved response in NH_3_ gas sensing tests compared with the pristine G channel. Moreover, the OP-G/G channel showed faster response and recovering process than the OP-G channel. Since this kind of composite channel is fabricated from chemical vapor deposited graphene and patterned with standard photolithography, the device dimension was much smaller than a gas sensor fabricated from reduced graphene oxide and it is favorable for the integration of a large number of sensing units.

## 1. Introduction

Gas detection and recognition is very important in both industry and daily life applications. Especially with the development of the Internet of things (IoT), the demand for miniaturized gas sensors with low power consumption will increase dramatically. In addition to specific gas detection, mixed gas component recognition will be required for many emerging IoT applications such as food quality monitoring [1,2], daily chemical industry [3,4,5] and agricultural products [6,7,8]. One of the basic mixed gas component recognition techniques is electronic nose (e-nose) [1,6,7], which is based on an array of gas sensors with different gas sensing characteristics and data processing with a neural network algorithm. An E-nose based solid state device has an advantage in miniaturization. However, commercial solid state e-nose is normally based on metal oxide sensitive material and usually needs to be heated up to over two hundred centigrade during sensing [1,6,7]. This demands huge power consumption and limits the number of gas sensing elements in the e-nose. Nanomaterials provide numerous opportunities to develop ultrasensitive gas sensors with room temperature operation and low power consumption [9,10,11]. Graphene is one of the most promising nanomaterials for gas sensing and attracts intense study due to its ultrahigh specific surface, ultrahigh carrier mobility and low electrical noise [12,13,14]. More importantly, graphene gas sensors operate at room temperature and graphene film is compatible with standard semiconductor fabrication processes [10,11,15]. These two features render graphene an ideal candidate material towards an integrated gas sensor array for e-nose applications.

Though pristine graphene (G) has demonstrated high sensitivity in several reports [13,16,17], its self-closed super-pi bond limits the molecular adsorption on the surface and thus its sensitivity in gas sensing [17]. Moreover, molecular adsorption on G is mainly based on Van der Waals interaction, which provides poor specificity. To address this issue, nanoparticle decoration and chemical treatment are widely explored to enhance both the sensitivity and specificity in gas sensing [10,11,18,19,20,21]. Those procedures introduce functional groups or defects on the graphene surface and provide effective molecule adsorption sites. However, this is at the cost of carrier mobility degradation. The carrier mobility degradation in turn reduces the sensitivity since the conductance of a channel is determined by both the carrier concentration and the carrier mobility. Increasing the effective adsorption sites while maintaining high carrier mobility is one of the major challenges, we are facing in the development of a graphene gas sensor. To address this challenge, here we propose a gas sensing channel with composite double layer graphene: a layer of pristine graphene at the bottom and a layer of functionalized graphene on the top. The functionalized graphene on the top provides adsorption sites while the G beneath serves as a fast carrier transport path.

A straightforward way is using reduced graphene oxide (rGO) as the functionalized graphene on the top since the high sensitivity of rGO in gas sensing is widely reported [10,11,15,20]. The residual oxygen functional groups such as carboxyl, hydroxyl and epoxy in rGO provide effective adsorption sites. However, rGO is not suitable for the fabrication of a micron-sized conductive channel, because the flake size of rGO reduced from GO is normally around several microns. An rGO conductive channel is layers of randomly stacked rGO flakes obtained on an interdigital electrode by a dip-and-dry process [11,15,20]. The uniformity of such a conductive channel could be obtained only on the scale much larger than the size of an individual rGO flake; for instance, hundreds of microns. Such a large channel size causes large power consumption and limits the device integration. To obtain a proposed composite double layer graphene channel of micron size, functionalization of large area graphene for top–down patterning process is required. In situ oxidation of chemical vapor deposited (CVD) graphene through a pure physical process such as thermal or plasma treatment is more appealing for micro-sized channel fabrication [22,23,24,25,26]. In this work, the functionalization of CVD graphene by using oxygen plasma is investigated. The oxygen plasma-treated graphene (OP-G) is used as the functionalized graphene on the top of the composite channel. The ammonia gas sensing performance of the developed composite channel (OP-G/G) is studied by comparing with a mono layer OP-G and a mono layer G channel. The results of our work show the OP-G/G composite channel has significantly improved sensitivity compared with pristine G and much faster response and recovering process compared with a mono layer OP-G channel.

## 2. Materials and Methods

### 2.1. CVD Graphene Preparation and Oxygen Plasma Treatment

The graphene film used in this study was grown by CVD method using methane (CH_4_) as carbon source and 25 μm thick copper foil (99.8% metals basis, purchased from Alfa Aesar Co., Ltd. Tianjing, China) as substrate. The detailed graphene preparation process is as follows: The copper foil was first ultrasonically cleaned in ethyl alcohol, then soaked into a diluted HCl solution to remove the surface oxides and finally rinsed several times by deionized water and dried with blowing N_2_. The copper foil was then mounted in the CVD furnace tube and annealed for 30 min at 1060 °C, under the flow of 100 sccm Ar. After that, 10 sccm CH_4_ carried by constant flow of 100 sccm H_2_ was fed into the reaction tube at the same temperature for the graphene growth. After 60 min, the furnace was shut down and rapidly cooled to room temperature in 2 min. As-grown graphene on copper foil was then put in a reactive ion etching (RIE) chamber for oxygen plasma treatment.

### 2.2. Sensor Fabrication

**Figure 1:**
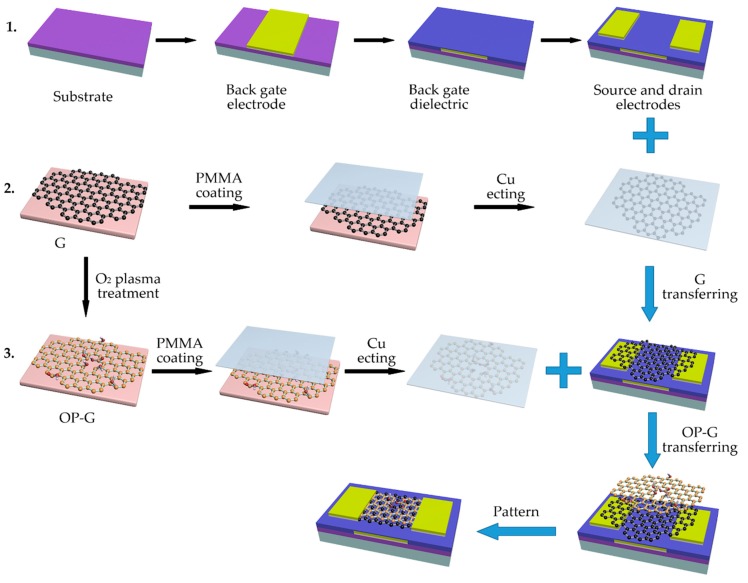
Schematic of the sensor fabrication process.

Figure 1 depicts the fabrication process of the OP-G/G composite channel based gas sensor. A Si wafer with a 300 nm silicon dioxide (SiO_2_) layer was used as substrate. 10 nm Ti and 50 nm Pd layer was deposited on the substrate using magnetron sputtering with a metal shadow mask to form a back gate electrode. Then, a 150 nm Al_2_O_3_ layer was deposited to form a back gate dielectric, followed by annealing at 800 °C to improve the quality of the dielectric layer. A 10 nm Ti layer and a 100 nm Pd layer were deposited on the Al_2_O_3_ layer to form the source and drain electrodes. The second line of the illustration in Figure 1 depicts the process of OP-G preparation and transfer. Both as-grown and oxygen plasma-treated graphene were transferred with Polymethyl Methacrylate (PMMA) as transfer vehicle and a (NH_4_)_2_S_2_O_8_ solution as copper foil etching agent. To remove the residual PMMA on the surface of the graphene films, the device was annealed under the protection of the flow of Ar and H_2_ after each transfer process. Finally, the composite graphene channel was patterned into a channel of 50 μm in width and 60 μm in length using photolithography.

### 2.3. Measurement Setup

**Figure 2:**
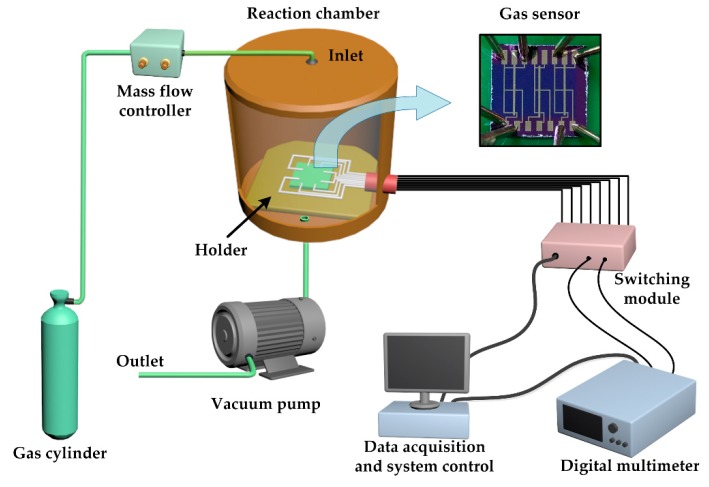
Schematic of the measurement setup.

Figure 2 displays the schematic of the gas sensing measurement setup. The sensor was placed in a custom reaction chamber with a volume of 10 L. The inlet of the chamber was connected to gas cylinders through mass flow controllers and the outlet was connected to a vacuum pump. The reaction chamber was first filled with dry air and then NH_3_ (99.99% purified) was fed into the chamber. The concentration of NH_3_ was controlled by gas flow, which was controlled by mass flow meter and feeding time. The feeding time was less than 16 s. After the gas sensing test, the chamber was opened and purged with air. The response of the device was measured with a digital multimeter (Keithley 2000). All the tests were carried out at room temperature.

### 2.4. Characterization Techniques

Field emission scanning electron microscopy (FESEM, GeminiSEM 500, Zeiss, Germany) was used to investigate the morphology of prepared samples. X-ray photoelectron spectroscopy (XPS, ESCALAB Xi+, Thermo Fisher Scientific, Massachusetts, America) analysis was performed to investigate elemental composition of the samples before and after oxygen plasma treatment. Raman spectra (Laser Raman Spectrometer, HONG KONG, China) was recorded using a 532 nm exciting laser.

## 3. Results

### 3.1. Characterization of G and OP-G

**Figure 3:**
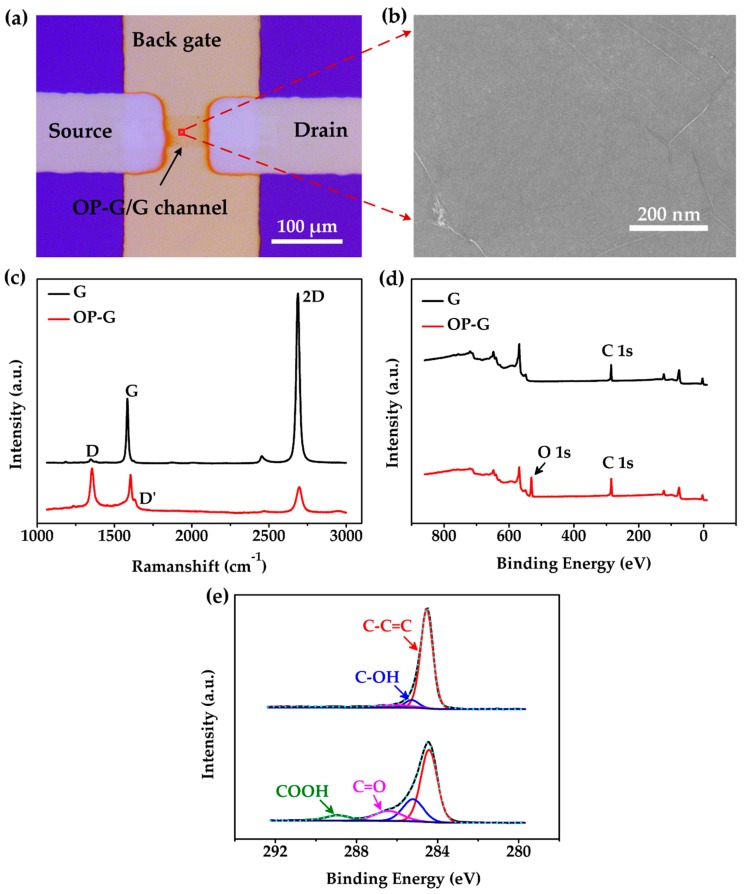
Material characterization. (**a**) Optical microscope image of the composite OP-G/G channel. (**b**) The FESEM image of the channel at the red square highlighted area of (a). (**c**) Raman spectra of pristine graphene (G) and oxygen plasma-treated graphene (OP-G) samples. (**d**) XPS survey spectra and (**e**) zoom-in plot and fittings of C 1s peak in XPS spectra.

The optical microscope image of the channel area of our device is displayed in Figure 3a. It shows a graphene ribbon which connects the source and drain electrodes. The back gate electrode was designed for a future gate voltage tunable gas sensing test. However, the sensor was only tested as a resistor in this work. Figure 3b shows an FESEM image of the graphene channel. The surface of the channel was very clean and flat excepting a few wrinkles and cracks [27].

Raman spectra of graphene before and after oxygen plasma treatment are shown in Figure 3c. Before oxygen plasma treatment, the negligible D peak indicated the high crystal quality of the pristine graphene. The intensity ratio of I_2D_/I_G_ was approximately 2.4, which confirmed that the pristine graphene was monolayer [28]. However, after oxygen plasma treatment, a distinct D peak at approximately 1351 cm^−1^ was observed and the amplitude of the 2D peak was significantly reduced alone with a shift of the G peak from 1590 cm^−1^ to 1604 cm^−1^. These results indicated a significant increase of carbon vacancies in the graphene. Furthermore, a D’ peak was found at approximately 1633 cm^−1^, which was related with more edges or defects in graphene film. The Raman spectra confirmed a successful introduction of defects or edges in the graphene lattice after oxygen plasma treatment.

XPS was further used to study the chemical status of graphene after oxygen plasma treatment. The results are also shown in Figure 3. In Figure 3d, no obvious O 1s peak was found in pristine graphene. To obtain detailed information of the bonds in graphene, the C 1s peak at 284.5 eV was analyzed by XPSPEAK software and the results are shown in Figure 3e. C 1s peaks were split into four peaks at binding energy of 284.42 eV, 285.21 eV, 286.52 eV and 288.95 eV, which correspond to C–C=C, C–OH, C=O and COOH, respectively [29]. The percentages of different C bonds were calculated by XPSPEAK software from C 1s peak. The results are shown in Table 1. The oxygen functional groups in OP-G were significantly larger than that in G. This indicated that oxygen groups were successfully introduced into graphene lattice by oxygen plasma treatment [22]. As shown in Table 1, the proportion of C–C=C remarkably decreased from 93.05% to 58.04%, while the proportions of bonds between carbon and oxygen in forms of C–OH, C=O and COOH are all increased. The proportion of C–OH significantly increased from 4.41% to 22.02%. The proportion of C=O increased from 1.89% to 13.37%, while the proportion of COOH increased from 0.65% to 6.58%. This result indicated an effective graphene decoration with oxygen functional groups after oxygen plasma treatment.

### 3.2. Gas Sensing Experiment

**Figure 4:**
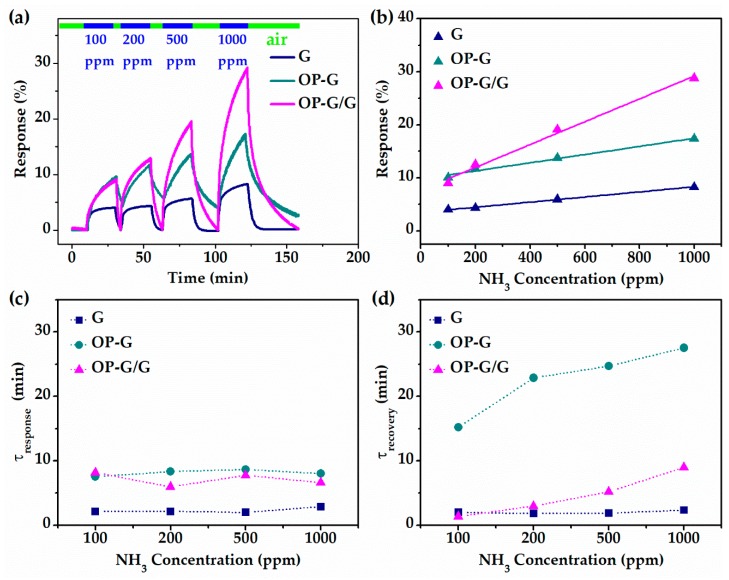
The relative NH_3_ gas sensing test results of G, OP-G and OP-G/G based sensors. (**a**) Response curves at different concentrations of NH_3_. (**b**) Sensitivity of three gas sensors. (**c**) Response and (**d**) recovery time constant of three gas sensors.

The respective responses of the sensors with G, OP-G and OP-G/G channels to NH_3_ are shown in Figure 4a. The response of the sensor is defined as follows:

$$\left[\left(R-R_{0}\right)/R_{0}\right]\times 100\%,$$

where *R*_0_ is the original resistance of the channel before the introduction of NH_3_ gas. The response of the sensor with the G channel was significantly lower than that of the sensor with the OP-G or OP-G/G channels for all of the tested ammonia concentrations. At 100 and 200 ppm NH_3_, the sensor with the OP-G channel and the sensor with the OP-G/G channel exhibited similar responses. However, when NH_3_ concentration increased to 500 and 1000 ppm, the response of the sensor with the OP-G/G channel was significantly better than the sensor with the OP-G channel. The responses of the three sensors after exposure to ammonia for 20 min are plotted in Figure 4b. All three sensors showed good linearity in response to NH_3_ within the concentration range from 100 to 1000 ppm. For 1000 ppm NH_3_, the response of the sensors with G, OP-G and OP-G/G channels are 7.6, 17.3% and 29.3%, respectively. The sensitivity of the three sensors was estimated from the linear curves in Figure 4b. The results showed that the sensor with OP-G/G channels had 6 and 2.8 times the sensitivity of sensors with G and OP-G channels, respectively. Thus, the sensor with the OP-G/G channel demonstrated the best performance among the three tested devices in terms of response and sensitivity.

The improved response of the sensors with the OP-G and OP-G/G channels may be attributed to two reasons. First, the oxygen functional groups introduced on the graphene surface provided more effective adsorption sites [30]. Second, the significantly enhanced *p*-doping level of OP-G and OP-G/G [31,32] increased the average amount of charge transferred from an adsorbed ammonia molecule to the channel. Therefore, the response of the sensors with OP-G and OP-G/G channels are both higher than the sensor with the G channel [33]. More importantly, the sensor with the composite OP-G/G channel showed significantly higher response than that with the mono OP-G channel. This may be attributed to a faster carrier transfer path beneath.

The response and recovery times are two other important figures of merit for gas sensors. The response and recovery time constants were extracted by exponential fitting of the response and recovery curves. The results are plotted in Figure 4c,d. The sensor with the G channel demonstrated the shortest response and recovery time, while the sensor with the mono OP-G channel showed the longest response and recovery time. The improvement of the sensitivity of OP-G was at a cost of sacrificing the response and recovery speed. However, the composite OP-G/G channel reported here demonstrated a faster response and recovery process compared with the mono OP-G channel. These results suggest that the sensor with the OP-G/G channel not only showed the highest sensitivity, but also partially maintained the fast response and recovery characteristics of the sensor with the G channel.

However, it is notable that the response curves of the OP-G and OP-G/G based sensors showed no stabilization within 20 min though the responses were already significantly larger than that of the G based sensor, which had already shown clear stabilization. When the detection step was increased to two hours, there was still no obvious stabilization for the OP-G and OP-G/G based sensors, as shown in Figure S1 in the Supplementary Information. Only double exponential function gives good fitting of the response curves of OP-G and OP-G/G based sensors. Double exponential function fitting is widely reported for the response of graphene and carbon nanotube based gas sensors [34,35,36]. It indicates that the response comprises a fast process and a slow process. The results in this report indicate that the slow process makes negligible contribution to the response of a G based sensor but significant contribution to the response of OP-G and OP-G/G based sensors. 

It is normally believed that the fast response is related with the adsorption on the graphene surface, while the slow process is related with the adsorption beneath the graphene at the interface with substrate [35]. However, this mechanism alone could hardly give a reasonable explanation for the significantly different contribution of the slow process in the G based sensor and the other two sensors. A possible understanding may find a different dynamic process for the adsorption on non-defective sites and that via oxygen functional groups. The adsorption on non-defective sites is mainly through Van der Waals force due to the self-closed super-pi bond of graphene [17]. It is a straightforward process with lower activation energy. Such adsorption contributes to the fast process. The adsorption via an oxygen functional group needs a surface transport before it reaches the oxygen functional group on OP-G. Moreover, the adsorption is through much strong interaction such as H- bond [37] and the activation energy for such absorption is larger than that of pure Van der Waals force. Such adsorption contributes to the slow process. It is similar for the recovery process. However, why the composite OP-G/G channel showed much faster recovery process compared with the mono OP-G channel is still not clear.

The OP-G/G based sensor was further tested with ammonia concentration down to 20 ppm. The response versus NH_3_ concentration (from 20 to 100 ppm) is plotted and shown in Figure S2 in Supplementary Information. The limit of detection (LOD) calculated by Equation S-3 was about 0.76 ppm (corresponding to a signal-to-noise ratio equals three) [38,39,40,41]. The performance of the sensor in this work was also compared with other types of NH_3_ sensors based on decorated graphene, RGO, metal oxide (MO_X_), etc. in Table S1 in Supplementary Information [42,43,44,45,46,47,48,49]. As shown in Table S1, our OP-G/G based sensor showed improved performance in response and recovery compared with graphene based NH_3_ sensors in other reports. The sensor in this report is even comparable with some MO_X_ based NH_3_ sensors in terms of LOD, response and recovery time.

To further explore the effect of humidity in NH_3_ test, the G, OP-G and OP-G/G based sensors were tested to 1000 ppm NH_3_ with different relative humidity (RH): 0%, 40% and 80%. The result is shown in Figure S3 in the Supplementary Information. The humidity enhanced the response of all three sensors. When the relative humidity increased from 0% to 80%, the response of G, OP-G and OP-G/G based sensor was increased by 8.5%, 8.8% and 16.1%, respectively.

**Figure 5:**
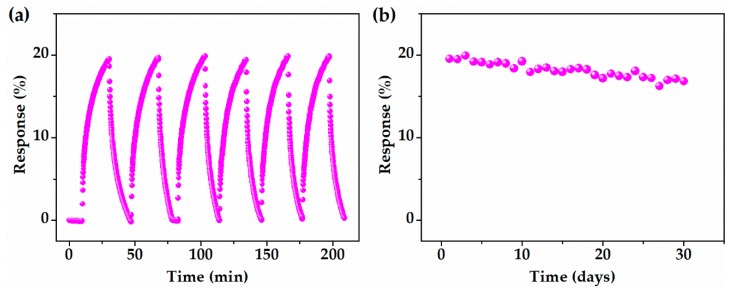
OP-G/G based sensor upon exposure to 500 ppm NH_3_ at room temperature: (**a**) The reproducibility and (**b**) long term stability.

The reproducibility and stability of the sensor with the OP-G/G channel was tested and the results are shown in Figure 5. As shown in Figure 5a, the sensor with the OP-G/G channel was repeatedly exposed to 500 ppm NH_3_ for 20 min. The sensor demonstrated a pretty good reproducibility. The relative standard deviation of the response in the six circles was within 1%. To further investigate the stability of the sensor with the OP-G/G channel, it was exposed to 500 ppm NH_3_ for 20 min each day within a month. The response was recorded and plotted in Figure 5b. The response of the sensor degraded from 19.5% to 16.8% after 30 days.

**Figure 6:**
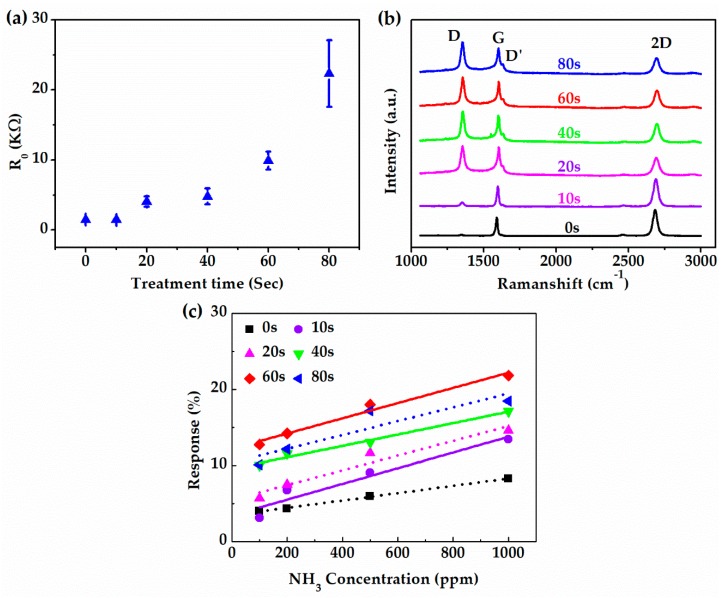
The effect of oxygen plasma treatment time. (**a**) Resistance and (**b**) Raman spectra of OP-G with different treatment time. (**c**) Sensitivity of OP-G with different treatment time towards different concentrations of NH_3_.

Oxygen plasma treatment time is critical for the quality of OP-G. In order to obtain the optimal oxygen plasma treatment time, a bunch of samples were treated for 10, 20, 40, 60 and 80 s, respectively. The resistance of OP-G with different treatment time is plotted in Figure 6a. The resistance increased with the increase of the treatment time. Especially, when the treatment time increased to 80 s, the resistance soared to more than 20 times that of pristine graphene. When the treatment time further increased to over 100 s, the resistance sharply increased to more than mega Ohm, which indicated a completely breaking of the graphene conductive channel. Figure 6b demonstrates the Raman spectra of the OP-G samples after different time of treatment. As the treatment time increases, D peak increased considerably accompanied by the appearance of a noticeable D’ peak, while the 2D peak decreased and broadened gradually with the increasing treatment time. The Raman spectrum result indicated that the defect density increased with the treatment time [32,50,51]. This is consistent with the resistance change shown in Figure 6a. The gas sensing response of the sensor with the OP-G channel with different treatment time is illustrated in Figure 6c. As the treatment time increased from 0 to 60 s, the response of the sensor increased in the whole range of NH_3_ concentration from 100 to 1000 ppm. However, as the treatment time further increased to 80 s, the response decreased. In conclusion, the OP-G with a treatment time of 60 s showed the best gas sensing performance (22.1% to 1000 ppm NH_3_). Thus, we chose 60 s as the treatment time for the preparation of the OP-G/G composite channel in this study.

## 4. Conclusions

In conclusion, a composite channel with OP-G on top and pristine graphene beneath was prepared from CVD graphene film and tested as a gas sensing material. In the NH_3_ gas sensing test, the sensor with the OP-G/G channel demonstrated the best performance in terms of both response and sensitivity. For instance, the NH_3_ response of the sensor with the OP-G/G channel was 4 times of the sensor with the pristine graphene channel and 1.7 times of the sensor with the mono OP-G channel, when the sensors were exposure to 1000 ppm NH_3_ at room temperature. The NH_3_ sensitivity of the sensor with OP-G/G channel was 6 and 2.8 times more than that of sensors with the G and OP-G channels, respectively. Moreover, the sensor with the OP-G/G channel also exhibited faster response and recovery process compared with the sensor with the OP-G channel. The significantly improved gas sensing performance is attributed to the cooperation effect between OP-G on top, which provides plenty of adsorption sites, and pristine graphene beneath, which provides a fast carrier transfer path. Finally, the optimal oxygen plasma treatment time is about 60 s in our experiment condition.

## Figures and Tables

**Table 1:** XPS analysis of C 1s peak of G and OP-G.

Bands	G	OP-G
C–C=C (284.42 eV)	93.05%	58.04%
C–OH (285.21 eV)	4.41%	22.02%
C=O (286.52 eV)	1.89%	13.37%
COOH (288.95 eV)	0.65%	6.58%

## Supplementary Materials

The following are available online at http://www.mdpi.com/1424-8220/19/3/625/s1, Figure S1: title, Table S1: title, Video S1: title.
