# Supplementary material for: A Gas Sensing Channel Composited with Pristine and Oxygen Plasma-Treated Graphene
Source: Sensors (Basel). 2019 Feb 1;19(3):625. doi: 10.3390/s19030625 (PMC6387050; doi:10.3390/s19030625)
Supplement: Supplementary file 1 [file sensors-19-00625-s001.pdf]

Supplementary Information

A Gas Sensing Channel Composited with Pristine and Oxygen Plasma-Treated Graphene

Haiyang Wu [1], Xiangrui Bu [1], Minming Deng [2], Guangbing Chen [2], Guohe Zhang [1], Xin Li [1,3], Xiaoli Wang [1,4] and Weihua Liu [1,5,6,*]

[1] School of Microelectronics, School of Electronics and Information Engineering, Xi'an Jiaotong University, Xi'an 710049, China; wuhaiyang@stu.xjtu.edu.cn (H.W.); bxr1212@stu.xjtu.edu.cn (X.B.); zhangguohe@xjtu.edu.cn (G.Z.); lx@mail.xjtu.edu.cn (X.L.); xlwang@mail.xjtu.edu.cn (X.W.)

[2] Science and Technology on Analog Integrated Circuit Laboratory, Chongqing 401332, China; yangjing@cetccq.com.cn (M.D.); cgb@sisc.com.cn (G.C.)

[3] Guangdong Shunde Xi'an Jiaotong University Academy, NO.3 Deshengdong Road, Daliang, Shunde District, Foshan 528300, China

[4] School of Science, Xi'an Jiaotong University, Xi'an 710049, China

[5] Key Laboratory for Physical Electronics and Devices of the Ministry of Education, Department of Electronic Science and Technology, School of Electronic and Information Engineering, Xi'an Jiaotong University, 28 West Xianning Road, Xi'an 710049, China

[6] Research institute of Xi'an Jiaotong University (Zhejiang), Hangzhou, Zhejiang 311215, China

* Correspondence:Correspondence: lwhua@mail.xjtu.edu.cn; Tel.: +86-29-8266-3343

The limit of detection (LOD) calculation

Due to the limitations of the gas distribution system in this work, it is hard to get the detection limit of NH_3 gas. But the theoretical achievable LOD value (corresponding to signal-to-noise equals 3) can be calculated [38]. The OP-G/G based sensor noise can be calculated using the relative resistance change from the baseline, which can be described as following equation [39]:

$$rms_{noise} = \sqrt{\frac{\sum_{i=1}^{N}(y_i - y)^2}{N}} \quad \text{(S-1)}$$

where y_i is measured data and y is the corresponding value from an exponential fit, N is the number of data.

Signal-to-noise (SNR) can be calculated by dividing the gas response by the sensor noise [40] as following equation:

$$SNR = \frac{S}{rms_{noise}} \quad \text{(S-2)}$$

where S is NH_3 gas response for the OP-G/G based sensor.

The theoretical measureable LOD (corresponding to a SNR of 3) for the OP-G/G based sensor can be calculated by [41]

$$x_{LOD} = \frac{3rms_{noise}}{slope} \quad \text{(S-3)}$$

In this work, we used 126 points in the baseline in Figure S2 (a) to derive the sensor noise. The noise is 0.00022 for the OP-G/G based sensor. The SNR is 147 for the sensor exposure to 20 ppm NH_3. The LOD (corresponding to a SNR of 3) is 0.76 ppm calculated by using Equation S-3.

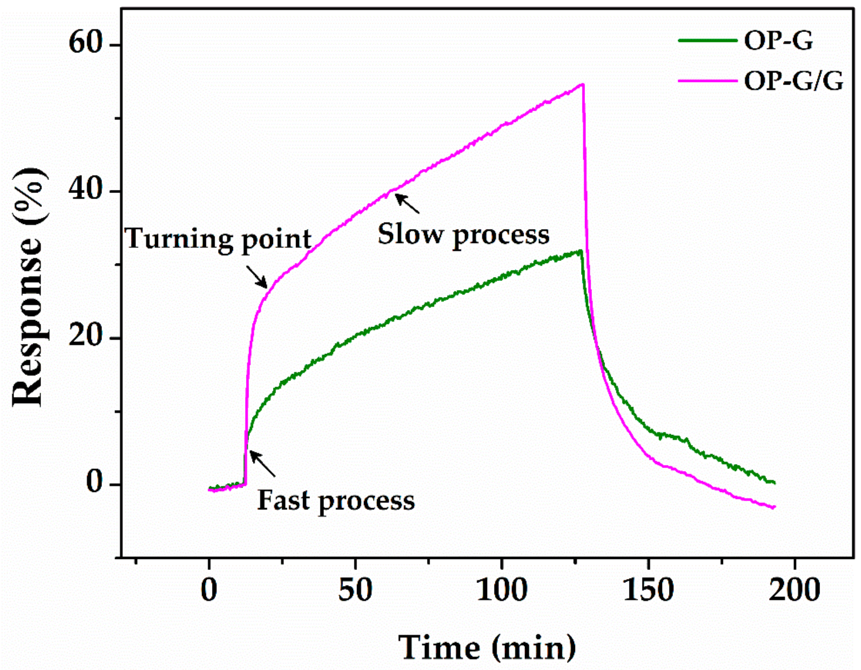

Figure S1. The response curve of OP-G and OP-G/G sensor tested in 1000 ppm NH_3 for 2 hours.

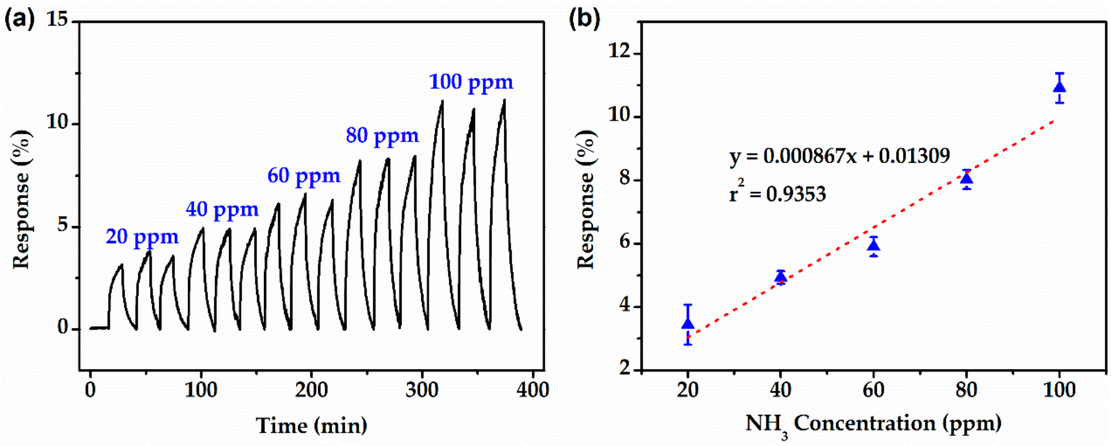

Figure S2. (a) The response curve of OP-G/G based sensor to low concentration of NH_3. (b) The linear fitting to the response of OP-G/G based sensor after 800 s exposure in different concentrations of NH_3.

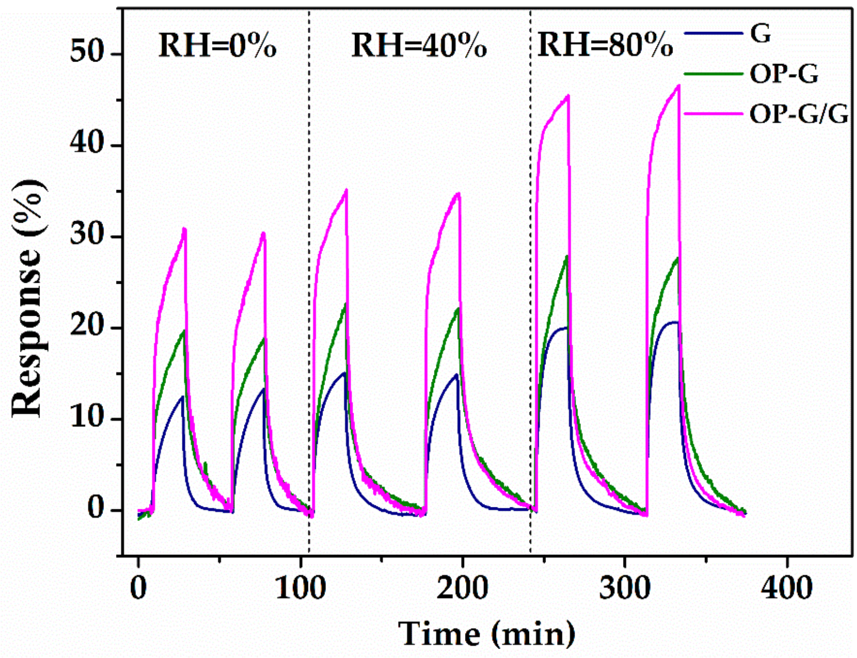

Figure S3. The gas sensing test to 1000 ppm NH_3 with different RH: 0%, 40% and 80%.

Table S1. Comparison of performances between different NH_3 sensors.

Materials	Handling method	Concentration of NH_3 (ppm)	Detecting temperature	Response ($\Delta R/R_0$) (%)	LOD (ppm)	Testing time (min)	Recovery Time (min)	Ref.
CVD graphene	OP-G/G	1000<br>100	RT<br>RT	29.3<br>4.1	<20	20<br>2	38<br>1.33	This work
CVD graphene	Aluminum oxide substrate	1300	RT	1.5	-	2.6	2.2	30
CVD graphene	Ag decoration	500	RT	9	-	10	-	31
CVD graphene	Pd decoration	100	150	2	-	5	>20	32
RGO	Pt decoration	1000	RT	10	-	2.7	4.8	33
GO	RGO/3-CuPc hybrids	3200	RT	15.4	-	15	>80	34
GO	P doping	100	RT	5.5	-	2.2	13.6	35
$NiO–SnO_2$	-	100	220	95	10	5.8	5.8	36
Pd-ZnO	-	100	210	85	5	1	1.33	37
